# A rare case report of iatrogenic Cushing syndrome with multisystem involvement due to prolonged steroid injections: a diagnostic challenge

**DOI:** 10.1097/MS9.0000000000004498

**Published:** 2025-12-04

**Authors:** Tayyeb Ali, Muhammad Hassan Javaid, Muddassir Khalid, Mehzad Javed, Mamoosa Zeb, Sadia Afridi, Kainat Bangash

**Affiliations:** a Department of Medicine, Gomal Medical College, Dera Ismail Khan, Pakistan; b Department of Medicine, Shifa College of Medicine, Islamabad, Pakistan; c Department of Medicine, Nishtar Medical University, Multan, Pakistan; d Department of Medicine, Khyber Girls Medical College, Peshawar, Pakistan

**Keywords:** cortisol, diabetes mellitus, hypothalamic–pituitary–adrenal axis suppression, iatrogenic Cushing syndrome, steroid misuse

## Abstract

**Introduction and importance:**

Iatrogenic Cushing syndrome is a multisystemic endocrinological disorder. It is caused by prolonged exposure to exogenous corticosteroids and remains an underdiagnosed endocrine disorder, particularly in low-resource settings where unsupervised self-administration and misuse of Over The Counter (OTC) steroid injections is common.

**Case presentation:**

We present a 57-year-old woman with a 20-year history of type 2 diabetes mellitus who presented to the notice of a clinician with multifocal recurrent left arm, leg, and breast cellulitis; diarrhea; bilateral pedal edema; and atrial fibrillation. The history of the patient was long-term treatment with insulin and frequent self-administered steroid injections for self-treated musculoskeletal pain, not prescribed. Despite classic Cushingoid features, her laboratory findings paradoxically revealed hypocortisolism and suppressed gonadotropins, consistent with hypothalamic pituitary adrenal (HPA) axis suppression. Management consisted of steroid withdrawal under observation, broad-spectrum antibiotics in cellulitis, hyperglycemia control with aggressive insulin, and supportive care.

**Clinical discussion:**

This case demonstrates the diagnostic challenge of exogenous Cushing syndrome, in which biochemical findings of low cortisol can mask clinical hypercortisolism. It underscores the systemic complications which include metabolic decompensation, infection susceptibility, and cardiovascular disease due to unregulated steroid use. Of interest is that the case demonstrates the public health significance of uncontrolled steroid access in low- and middle-income nations like Pakistan, where over-the-counter misuse mainly contributes to underdiagnosed iatrogenic Cushing syndrome

**Conclusion:**

Clinicians should consider iatrogenic Cushing syndrome in patients with unexplained multisystem problems. Regulatory control of over-the-counter steroid access and patient education are crucial to preventing such cases.

## Introduction

Cushing Syndrome is a rare multisystemic endocrine disorder that occurs due to chronic exposure to high blood cortisol levels^[1]^. Cushing syndrome is commonly caused by administration of supraphysiologic doses of corticosteroid medications (iatrogenic Cushing syndrome) and less commonly by excessive production of corticosteroids by the adrenal cortex (endogenous Cushing syndrome)^[2]^. Iatrogenic Cushing Syndrome is underdiagnosed primarily in low-resource settings where over-the-counter steroid medications and injections, mostly dexamethasone, are frequently used without seeking medical supervision. This case report presents a case of a 57-year-old female with multifocal cellulitis, Atrial fibrillation, Bilateral pedal edema, diarrhea, and a medical history of diabetes mellitus type 2 for 20 years and a drug history of insulin and corticosteroid medications, most likely steroid painkiller injections^[3]^. The case is rare and noteworthy not only because of the atypical presentation of multifocal cellulitis, but also because it highlights the importance of recognizing all multisystemic symptoms when making a diagnosis, as well as taking a detailed drug history^[4]^. This case also has a public health implication in regulating the unregulated use of over-the-counter steroid medications in low-resource settings. A community pharmacy simulation study in Pakistan found that 15.1% of cases presented to pharmacies resulted in a steroid being dispensed without a proper prescription^[5]^. As per our knowledge, very few or no cases with such multisystem involvement were reported in literature.

**HIGHLIGHTS**

- Iatrogenic Cushing Syndrome is often underdiagnosed in low-resource settings.
- Prolonged OTC steroid injections led to multisystem complications in this patient.
- Case presented with atypical recurrent multifocal cellulitis and atrial fibrillation.
- Clinical hypercortisolism with paradoxical hypocortisolism added diagnostic dilemma.
- Highlights urgent need to regulate OTC steroid misuse in low- and middle-income countries

We, therefore, present a case of a 57-year-old female with a 20-year history of diabetes mellitus type 2 presented to the Outpatient Department (OPD) with recurrent cellulitis affecting the left leg, left arm, and left breast. The aim of this report is to present a rare and multifaceted case of iatrogenic Cushing syndrome with paradoxical hypocortisolism and recurrent multifocal cellulitis as well as to discuss diagnostic pitfalls that can occur from exogenous steroid use and to emphasize the public health implications of unregulated steroid access in low-middle-income countries.

‘This case report has been reported in line with the SCARE checklist [Kerwan A, Al-Jabir A, Mathew G, Sohrabi C, Rashid R, Franchi T, Nicola M, Agha M, Agha RA. Revised Surgical CAse Report (SCARE) guideline: An update for the age of Artificial Intelligence. Premier Journal of Science 2025:10;100 079].^[3]^

## Case presentation

**Figure 1.**
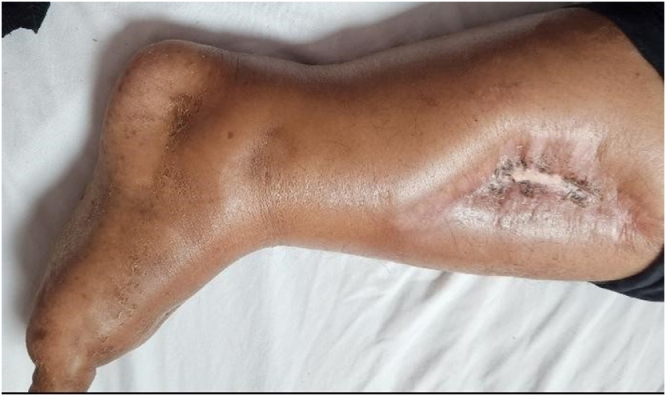
A healed cellulitis scar on the left leg (arrow).

A 57-year-old female with a 20-year history of Diabetes mellitus type 2 presented to the OPD with recurrent cellulitis affecting the left leg, left arm, and left breast. The first episode of cellulitis occurred 2 weeks prior on the left leg (as shown in Fig. 1 and Table 1) and has since occurred at multiple locations. She also reported loose stools for the past 2 days. The patient also had a drug history of Insulin for 10 years for the management of diabetes. The patient also received self-administering steroid injections for musculoskeletal pain, which likely contained corticosteroids for the past 1 year without prescription and medical supervision. The patient also had a family history of ischemic heart disease (IHD) and atrial fibrillation. The patient had a known history of diabetic foot ulcer.

**Table 1:** Timeline of case presentation

Date/Duration	Events/Findings	Interventions
1 year prior	Began unsupervised intramuscular steroid injections for musculoskeletal pain (every2–3 weeks)	No intervention; self administered
2 weeks before presentation	First episode of left leg cellulitis	Oral antibiotics (self-medication)
At presentation	Presented with recurrent cellulitis (arm, leg, breast), diarrhea, pedal edema	Admitted to the hospital
1-7 days post presentation	Initiated antibiotics, insulin adjustment, gradual steroid withdrawal	Gradual improvement in cellulitis and other symptoms
After 4 weeks	Outpatient follow-up; stable glycemia, no recurrence of cellulitis	Continued follow up

### Clinical findings

Physical examination of the patient revealed symptoms typical of Cushing Syndrome, including a puffy face, Cushingoid adiposity, multiple sites of cellulitic lesions on the skin, abdominal striae, a buffalo hump, and acanthosis Nigricans. The patient was also plethoric. On further investigation, the patient reported chronic fatigue, weight gain, and easy bruising. The patient was hypertensive, and the blood pressure was 150/90. Fasting blood glucose levels were also elevated. Her Glycated Haemoglobin (HbA1c) was also high, 12.7%, suggesting poorly controlled diabetes mellitus. The patient also had a fever, with a body temperature of 99 °F. Her pulse rate was 88 beats per minute (bpm). Her body mass index (BMI) was also elevated, and oral ulcers were also found.

### Investigations

**Figure 2.**
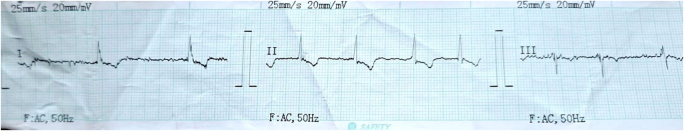
ECG showing inverted T waves indicating atrial fibrillation.

Laboratory investigations revealed subclinical hyperthyroidism with suppressed Thyroid Stimulating Hormone (TSH) and normal Thyroxine levels as shown in Table 2. Differential diagnoses included endogenous Cushing disease, metabolic syndrome, and diabetic dermopathy. Due to particular clinical presentations, biochemical lab findings, and history of exogenous steroid exposure, a clinical diagnosis of iatrogenic Cushing syndrome was made. An overnight dexamethasone suppression test was performed to exclude any cause of endogenous access of cortisol, which further confirmed the diagnosis. Due to the presence of pedal edema and a family history of heart disease, an electrocardiogram (ECG) was also performed, and the results showed elevated T waves which indicated atrial fibrillation as shown in Figure 2

**Table 2:** Summary of laboratory investigations

Tests	Results	Reference Range	Interpretation
Morning Serum Cortisol	5.2 µg/dL	7–25 µg/dL	Low
TSH	0.012 µIU/mL	0.5–5.0 µIU/m	Suppressed
FSH	0.565 mIU/mL	3–20 mIU/mL	Suppressed
ALP	245 U/L	44–147 U/L	Elevated
HbA1c	12.7%	<6.5 %	Poorly controlled
Dexamethasone Suppression Text	<1 µg/dL post-dexamethasone	<1.8 µg/dL (normal suppression)	Consistent with exogenous source

### Management

The patient was admitted to the endocrinology ward and initiated on broad-spectrum antibiotics for management of cellulitis, which occurred due to an immunosuppressed state because of the administration of exogenous steroids. Meropenem 1 g IV was administered three times daily, and metronidazole was administered three times a day. The reason for selecting broad-spectrum meropenem empirically was to prevent recurrent multifocal cellulitis. The therapy was later de-escalated based on clinical response.

For pain management, intravenous Tramadol (50 mg) diluted in 100 mL normal saline was administered slowly. Granisetron (1 mg) was co-administered to prevent opioid-induced nausea and vomiting because there was risk of gastrointestinal intolerance due to corticosteroid exposure and metabolic instability. Probenecid, a nonsteroidal anti-inflammatory drug (NSAID), was also provided as an additional analgesic. Intravenous paracetamol was also administered to reduce fever as well as pain. Steroid-induced hyperglycemia was managed by short-acting insulin (Actrapid) and long-acting insulin (Lantus), titrated according to capillary glucose levels. Gradual tapering of corticosteroids was implemented to avoid adrenal crisis, transitioning to physiological hydrocortisone replacement (10 mg AM, 5 mg PM) for 4 weeks under endocrinology supervision before discontinuation. For the management of steroid-induced Osteoporosis, oral calcium (Calcium Carbonate) and Vitamin D_3_ supplementation was initiated. High blood pressure was managed by administering a 5 mg tablet of bisoprolol once daily. For the management of loose motions and diarrhea, an Oral Rehydration Solution (ORS) was administered along with Loperamide. Supportive therapy included administration of Esomeprazole for protection of the gastrointestinal tract due to recurrent use of NSAIDs and steroids.

### Outcomes and follow-up

The patient was followed for 4 weeks post-discharge. The patient responded well to the above management. She reported complete resolution of cellulitis, improvement in glycemic control (HbA1c reduced to 8.9%), and normalization of cortisol levels.The steroid injections were stopped after effective patient counseling. The patient was then referred for physical therapy to manage musculoskeletal pain.

## Discussion

A literature search was performed in PubMed and Google Scholar (2013–2025) using the terms “iatrogenic Cushing syndrome,” “exogenous steroids,” “multifocal cellulitis,” and “HPA axis suppression.” Only English-language human case reports and reviews were included

Cushing syndrome usually presents with the classical features of moon face, central obesity, torso hump, purple striae, and hypertension or diabetes. However, its manifestation may sometimes take a rare or unique presentation, such as recurrent multifocal cellulitis, which is a very unusual manifestation of the disease. It reflects the excessive immunosuppression due to the misuse of OTC steroids. Though Cushing syndrome is a relatively common disease, its diagnosis, especially if it’s the iatrogenic Cushing syndrome, remains difficult^[6]^. It usually remains underdiagnosed in low-resource settings due to the misuse of over–the–counter (OTC) steroids^[7]^. The areas where the oral or injectable steroids are easily accessible and available to the public, even without prescriptions, are reporting high cases of iatrogenic Cushing syndrome. The unchecked and unregulated OTC steroids prevalence is reportedly being documented as the driver for iatrogenic cases^[8,9]^.

The patient from Pakistan, documented as the case of iatrogenic Cushing disease, is presented with an atypical presentation of the disease. Actually, repeated, prolonged, and unchecked chronic steroid use leads to an increased susceptibility to skin and soft-tissue infections^[10,11]^ due to immunosuppression, thus contributing to multifocal recurrent cellulitis (see Fig. 1). The steroid-related risk of opportunistic infections increases with the increased cumulative dose and longer duration of systemic glucocorticoids^[12]^. Despite the infection profile, the chronic use of steroids also worsens the glycemic control and precipitates diabetes. The basal-bolus insulin regimens are usually recommended for managing such metabolic complications of steroid misuse^[13]^. This patient witnessed the complex multi-system involvement, presenting with atrial fibrillation (see Fig. 2), hypertension, osteoporosis, hypogonadism, diabetes, and recurrent infections. Such endocrine disturbances, cardiovascular sequelae, and bone death effects are much anticipated outcomes in the iatrogenic Cushing syndrome^[14–16]^. Wound healing is also significantly impaired, as steroids disrupt innate and cellular immune responses, thereby increasing the severity of skin infections^[10]^. The diabetic foot ulcer, when combined with recurrent steroid-induced immunosuppression (SSTI), dramatically increases the risk of such an atypical presentation of multifocal cellulitis^[10,11]^. Furthermore, the hypocortisolism on the laboratory investigations adds to the diagnostic dilemma of the case^[17]^. This is due to paradoxically low morning cortisol levels, resulting from the suppression of ACTH and cortisol production, most likely caused by chronic exogenous steroid use. Thus, such a paradox, characterized by clinical features of hypercortisolism and laboratory investigations of hypocortisolism, makes the picture unclear^[17,18]^. The recommended tests for such cases include morning serum cortisol, late-night salivary cortisol, or a 24-hour urinary cortisol test, when the endogenous cause is suspected. Dexamethasone suppression tests are also indicated when needed. Additionally, cultures for local infections and imaging to rule out Cushing disease due to hypothalamic or pituitary causes^[19]^.

The management approaches for such a patient are supported by various guidelines. However, the abrupt withdrawal of corticosteroids is not recommended due to the risk of disturbance of the Hypothalamus-Pituitary Axis (HPA). Hence, a supervised taper and follow-up of HPA axis recovery is advised^[17,18]^. The cornerstone should be to treat infections aggressively with antibiotics. The empirical broad-spectrum regimen, which includes coverage for MRSA and gram-negative bacteria, is recommended. It includes vancomycin with piperacillin-tazobactam or carbapenem regimen as per guidelines^[10,11]^. The glycemic control should be concurrently balanced with insulin to improve results^[13]^. Patient education and counseling are mandatory to avoid recurrence. They should be educated about the risks of unregulated steroid use, either oral or injectable. Hence, removing access to OTC steroids is one of the key interventions to address the recurrent iatrogenic Cushing syndrome^[20]^. A vast literature gap remains to be covered regarding these types of cases. This case emphasizes the multifaceted and diagnostic difficulty of iatrogenic Cushing Syndrome, especially if multifocal cellulitis, panhypopituitarism, subclinical hyperthyroidism, and cardiac defects coexist in a case of long-standing diabetes. It emphasizes the importance of meticulous drug history taking, even more so in low-resource settings where corticosteroids are commonly dispensed without a medical prescription. The existing data is heavily reliant on case reports; therefore, gaps in reporting and surveillance need to be addressed as shown in Table 3

**Table 3:** Findings of case

Clinical Feature	Present in our case	Occurrence	Reference
Multifocal cellulitis	Yes	Rare	^[21]^
Hypocortisolism with cushingoid features	Yes	Occasional	^[13]^
Atrial fibrillation	Yes	Uncommon	^[22]^
OTC steroid misuse	Yes	Rising	^[3]^

The systematic data on frequency, outcomes, and drivers of OTC steroid misuse from low- and middle-income countries like Pakistan are lacking. Such steps and extended studies are needed for effective healthcare policy-making. There should be strict enforcement of regulations on steroid sales and checks and balances on the healthcare system in rural areas. The pharmacist’s stewardship should be encouraged. Additionally, the case highlights the necessity of public health programs to control the over-the-counter use of steroids and increase awareness among healthcare providers and the general public. The awareness should also be improved regarding the adverse effects of the misuse of steroids. Such case reports and instances from Pakistan and similar settings advocate the need for these steps. Early identification and proper management of steroid-induced complications can avoid significant morbidity.

This case report has few limitations which include incomplete microbiological culture data and short follow-up duration. However, complete clinical, laboratory, and therapeutic documentation provides valuable insight into this rare presentation.

This manuscript complies with TITAN Guidelines, 2025, declaring no use of AI (20).

## Conclusion

This case highlights the paradoxical coexistence of clinical hypercortisolism and biochemical hypocortisolism which can occur due to chronic exogenous steroid use. Clinicians should maintain vigilance for such diagnostic paradoxes, especially in patients with recurrent infections and metabolic instability. Stronger regulation of steroid sales and improved community education are essential to curb the growing burden of iatrogenic endocrinological disorders.

### Patient perspective

The patient expressed relief after learning that her long-term health problems were caused by the unsupervised use of steroid injections. She emphasized the importance of community awareness about the dangers of over-the-counter steroid use and expressed gratitude for counseling and follow-up support.

## Data Availability

Data are available on request from the authors.

## References

[1] Uwaifo GI Hura DE. Hypercortisolism [Internet]. Nih.gov. StatPearls Publishing. 2023. https://www.ncbi.nlm.nih.gov/books/NBK551526/

[2] Dunn C Amaya J Green P. A case of iatrogenic cushing’s syndrome following use of an over-the-counter arthritis supplement. In: Moore WV ed.. Case Reports in Endocrinology. 2023:1–3. 10.1155/2023/4769258 PMC10024620 36941974

[3] Ali M Abbasi BH Ahmad N. Over-the-counter medicines in Pakistan: misuse and overuse. Lancet 2020;395:116. 31929013 10.1016/S0140-6736(19)32999-X

[4] Furqan Khurshid H Zikria S Hamid S. Self-medication of corticosteroids: a life threatening case report from Pakistan. J Pharm Pract Community Med 2016;2:96–99.

[5] Gillani AH Arshad H Mujtaba H. Dispensing of antibiotics for tuberculosis patients using standardized patient approach at community pharmacies: results from a cross-sectional study in Pakistan. Front Public Health 2024;11:1241551. 38259789 10.3389/fpubh.2023.1241551 PMC10801376

[6] Chaaban S Sadikot RT. Bacterial infections associated with immunosuppressive agents commonly used in patients with interstitial lung diseases. Pathogens 2023;12:464. 36986386 10.3390/pathogens12030464 PMC10053664

[7] Burnham JP Kirby JP Kollef MH. Diagnosis and management of skin and soft tissue infections in the intensive care unit: a review. Intensive Care Med 2016;42:1899–911. 27699456 10.1007/s00134-016-4576-0 PMC6276373

[8] Chastain DB Spradlin M Ahmad H. Unintended consequences: risk of opportunistic infections associated with long-term glucocorticoid therapies in adults. Clin Infect Dis 2023;78:e37–e56. 10.1093/cid/ciad474 37669916

[9] Shah P Kalra S Yadav Y. Management of glucocorticoid-induced hyperglycemia. Diabetes Metab Syndr Obes: Targets Ther 2022;15:1577–88. 10.2147/DMSO.S330253 PMC9142341 35637859

[10] Lee Y Jehangir Q Poisson L. Abstract 13683: corticosteroids and the risk of atrial fibrillation in hospitalized COVID-19 patients. Circulation 2021;144.

[11] Mohammed A Mansour A Ahmed J. Effect of exogenous glucocorticoids on male hypogonadism. Biomed Rep 2020;13:12. 32765851 10.3892/br.2020.1319 PMC7391295

[12] Laurent MR Goemaere S Verroken C. Prevention and treatment of glucocorticoid-induced osteoporosis in adults: consensus recommendations from the belgian bone club. Front Endocrinol (Lausanne) 2022;13:908727. 35757436 10.3389/fendo.2022.908727 PMC9219603

[13] Matos AC Srirangalingam U Barry T. Cushing’s syndrome with low levels of serum cortisol: the role of inhaled steroids. Clin Med 2011;11:404–05. 10.7861/clinmedicine.11-4-404 PMC5873758 21853845

[14] Raveendran AV. Inhalational steroids and iatrogenic cushing’s syndrome. Open Respir Med J 2014;8:74. 25674177 10.2174/1874306401408010074 PMC4319196

[15] Anne T Iatrogenic cushing syndrome: practice essentials, frequency, mortality/morbidity [internet]. Medscape.com. Medscape. 2024 Accessed 2025 Sep 18. https://emedicine.medscape.com/article/117365-overview

[16] Shakeel S Nesar S Iffat W. Pharmacists’ insights and behaviors in preventing the misuse of topical corticosteroids in Pakistan: a mixed-method study. Cosmetics 2021;8:72.

[17] Zaveri D Thakkar M Solanki P. Dexamethasone induced cushing’s syndrome: a case report. Glob Acad J Pharm Drug Res 2022;4:73–75.

[18] Barbot M Zilio M Scaroni C. Cushing’s syndrome: overview of clinical presentation, diagnostic tools and complications. Best Pract Res Clin Endocrinol Metab 2020;34:101380. 32165101 10.1016/j.beem.2020.101380

[19] Berbudi A Rahmadika N Cahyadi AI. Type 2 diabetes and its impact on the immune system. Curr Diabetes Rev 2020;16:442–49. 31657690 10.2174/1573399815666191024085838 PMC7475801

[20] Kamel SI Rosas HG Gorbachova T. Local and systemic side effects of corticosteroid injections for musculoskeletal indications. AJR Am J Roentgenol 2024;222:e2330458. 38117096 10.2214/AJR.23.30458

[21] Chastain DB Spradlin M Ahmad H. Executive summary: state-of-the-art review: unintended consequences: risk of opportunistic infections associated with long-term glucocorticoid therapies in adults. Clin Infect Dis 2024;78:811–12. 38598566 10.1093/cid/ciae129

[22] van der Hooft CS Heeringa J Brusselle GG. Corticosteroids and the risk of atrial fibrillation. Arch Intern Med 2006;166:1016–20. 16682576 10.1001/archinte.166.9.1016

